# Supplementary material for: Are carbon emissions trading and green financial instruments synergistic? -Comprehensive quantitative research based on content analysis
Source: PLoS One. 2024 Mar 7;19(3):e0298601. doi: 10.1371/journal.pone.0298601 (PMC10919601; doi:10.1371/journal.pone.0298601)
Supplement: S1 Data — (ZIP) [file pone.0298601.s001.zip › Data/地级市Z_F工作报告.zip!/║╙▒▒/▓╫╓▌╩╨/└┤╫╘╣1⁄2╓┌║┼í╛┬φ┐╦╩2╛▌═°í┐_2011.docx]

各位代表：

从今年开始，我市将实施第十二个五年规划。按照市委七届十次全会通过的《中共沧州市委关于制定国民经济和社会发展第十二个五年规划的建议》，市政府制定了《沧州市国民经济和社会发展第十二个五年规划纲要（草案）》。现在，我代表市政府，向大会作报告，请各位代表连同《纲要（草案）》一并审议，并请市政协委员和列席会议的同志提出意见。

一、“十一五”时期经济社会发展成就

“十一五”时期，是我市经济社会发展进程中不平凡的五年。全市人民在省委、省政府和市委的坚强领导下，以科学发展观为统领，强力推进沿海强市建设，经济快速发展，社会持续进步，人民生活日益提高，圆满完成“十一五”确定的各项任务目标，开创了沧州经济社会发展的新局面。

——经济发展步入快车道，综合实力跃上新台阶。2010年，全市地区生产总值突破2000亿元大关，实际完成2203亿元，比2005年翻了一番，年均增长13.5%；全部财政收入完成271.2亿元，比2005年增长2.27倍，年均增长26.7%。一般预算收入完成91.3亿元，比2005年增长1.92倍，年均增长23.9%；规模以上工业增加值完成807.5亿元，比2005年增长1.13倍，年均增长16.3%；固定资产投资完成1448.1亿元，比2005年增长3.1倍，年均增长32.5%。社会消费品零售总额573亿元，比2005年增长1.3倍，年均增长18%。

——渤海新区建设全面启动，沿海经济发展实现新跨越。按照“从大运河走向渤海湾”的战略理念，加快沧州沿海地区开发开放，组建成立了沧州渤海新区，编制完成了港口、产业和城市发展等多项规划。黄骅综合大港一期工程如期完工，顺利通航。临港产业聚集步伐明显加快，谋划、建设亿元以上重点项目140个，总投资4500亿元。集疏运配套体系加快推进，16平方公里的新城起步区建设全面启动。2010年，渤海新区完成地区生产总值321亿元，比上年增长19.9%，财政收入52.5亿元，增长41.5%，发展的活力显著增强，成为沧州经济发展的新亮点。

——重点项目建设加快推进，产业结构调整取得新进展。“十一五”期间，全市在建和竣工投产投资超10亿元以上项目21个，100亿元以上项目5个。华北石化千万吨炼油等一批重点项目开工建设，沧州中铁二期、金牛化工离子膜烧碱等项目竣工投产，杨埕水库建成投用。产业结构调整步伐加快，石油化工、管道装备、冶金、机械制造、食品加工等传统产业进一步壮大。争创中国驰名商标7个。高新技术产业增加值占规模以上工业增加值的比重达到11.9%。开发区和工业聚集区建设扎实推进，沧州、任丘等4个开发区实现扩区，沧州高新技术产业开发区被批准为省级高新区，沧州临港化工园区升级为国家级经济技术开发区。到2010年，全市省级以上经济开发区和产业聚集区达到17个，入园企业5142家。

——基础设施建设日趋完善，城乡面貌呈现新变化。以港口、铁路、高速公路建设为重点，大力构筑现代综合交通体系。沧港、沧保、津汕、大广、沧京沧州段等5条（段）高速公路建成通车，新增通车里程327公里，总里程达到538公里。建设110千伏以上变电站32座，新增发电装机容量317万千瓦。深入开展城镇面貌三年大变样活动，全市累计拆迁拆违2311万平方米，其中，主城区15个城中村完成整体拆迁改造。沧州体育馆、国际会展中心、狮城公园等一批标志性建筑投入使用，新建五星级酒店2家、标准化菜市场26个。五年新建、打通、拓宽城市道路36条，环城高速公路建成通车。中心城区城市绿地面积达到1846公顷，建成区绿化覆盖率达到41%，人均公园面积由3平方米提高到10平方米，顺利通过省级园林城市验收。全市新增集中供热面积684万平方米。建设廉租住房5000套，人均住房面积达到33.3平方米，比2005年增加6.1平方米。以“十个一”工程为标志的县城建设步伐加快，累计完成投资246亿元。积极推动新民居示范工程建设，360个示范村完成规划，315个村开工建设。全市城镇化率达到45%，比2005年提高了9.6个百分点。

——体制机制不断创新，改革开放开创新局面。以产权制度改革为核心，扎实推进国有工商企业改革，企业发展活力明显增强。加快行政管理体制改革，政府职能进一步转变。农村综合改革、医药卫生体制改革、文化体制改革深入推进。财税金融改革取得重大突破，连续两年被省政府授予“金融生态市”荣誉称号。民生银行、交通银行、河北银行、华夏银行相继落户沧州，天交所渤海股权交易中心、河北恒银期货沧州营业部挂牌营业，沧州银行在全省率先晋升为地方商业银行二级行。组建了沧州建设投资公司和河北渤海投资公司，总资产分别达到120亿元和140亿元，境内外上市企业达到14家。对内对外开放迈出新步伐，成功举办中国吴桥国际杂技艺术节（沧州会场）和中国沧州国际武术节，扩大了沧州的知名度。加强与京津特别是央企的对接合作，积极引进战略投资者，与中石油、中海油、中石化等一批大型企业集团实现了战略合作，日本矢崎、香港联塑、法液空等一批境内外知名企业落户沧州。全市五年累计实际利用外资8.8亿美元，引进市外资金504亿元，外贸进出口总额完成73.8亿美元，经济外向度明显提高。

——县域经济发展提速，“三农”工作取得新成效。县域特色产业迅猛发展，年营业收入超10亿元的县域特色产业集群达到12个。涌现出肃宁—中国裘皮之都、孟村—中国弯头管件之都、泊头—中国铸造之乡、东光—中国包装机械之乡、盐山—中国管道装备制造基地等品牌县市。2010年，县域经济地区生产总值达到1500亿元，比2005年增长72.8%。18个县（市、区）全部财政收入均超两亿元，其中，6个县（市、区）超过10亿元，渤海新区和任丘市超过50亿元。认真落实各项强农惠农政策，全面取消农业税，累计发放粮食和农资直补等各类补贴25.2亿元。2010年粮食总产达到486万吨，连续7年丰产丰收，畜牧、蔬菜、棉花、果品生产稳步发展。产业化经营步伐不断加快，农业产业化率达到49.3%，比2005年提高6.8个百分点。五年来，共投入扶贫开发资金26.6亿元，52万人口实现稳定脱贫，农村劳动力转移就业87万人。加强农村道路、通讯等基础设施建设，新建农村道路4360公里，在全省率先实现了村村通油路、通电话、通宽带。积极开展农户科学储粮工程，推广小粮仓4.5万个。深入实施“万村千乡”市场工程，新建改建农家店6537个，农村流通网络进一步健全。

——节能减排工程扎实推进，环境质量得到新提升。深入实施十大节能工程和“8755”减排工程，预计可完成“十一五”节能减排目标。2010年单位生产总值能耗较2005年降低20%，化学需氧量、二氧化硫排放量分别削减18%和11.7%。全市15个污水处理项目和13个垃圾处理项目全部建成，城市生活垃圾无害化处理率、城市污水集中处理率和工业固体废物综合利用率分别达到100%、85%和99.5%，肃宁、沧县、沧州大化和沧州炼油厂顺利通过省“双三十”目标考核。大力实施“碧水蓝天”工程，加强重点领域生态环境保护，2010年市区空气质量二级以上天数达到335天，比2005年增加40天。

——民生工程成效显著，人民生活水平得到新提高。坚持以人为本，大力改善民生。2010年城镇居民人均可支配收入16116元，农民人均纯收入5528元，分别比2005年增加7519元和2217元，干部职工年人均工资收入由16132元提高到39600元。解决了248.6万城乡居民饮水安全问题。就业规模不断扩大，五年累计实现城镇就业20万人，城镇登记失业率控制在4.5%以内。社会保障体系进一步健全，城镇职工基本养老保险、医疗保险、工伤保险、生育保险、失业保险覆盖面不断扩大，城镇居民基本医疗保险全面实施，任丘、青县等5县（市）成为全国新型农村养老保险试点县。新建、改扩建敬老院、光荣院、福利院“三院”合一型民政事业服务中心18所，乡镇区域敬老院36所，集中供养能力明显提升。住房公积金累计归集完成68.5亿元，缴存覆盖率达到98.2%。新型农村合作医疗制度覆盖全市，参合率达到93.3%，农民群众看病难、看病贵问题得到进一步解决。

——各项社会事业协调发展，和谐沧州建设迈出新步伐。认真落实以县为主的九年义务教育管理体制，全面实现城乡免费义务教育，办学条件明显改善，五年新建、改扩建中小学230所，农村中小学全部实现集中供暖。《主城区教育专项规划》顺利实施，基础教育三年行动计划全面展开。中、高等教育结构不断完善，职业教育规模稳步扩大，沧州师专成功升本。公共卫生和基本医疗服务体系不断健全，全市改造乡镇卫生院175所，新建、改造村卫生室2943个，重大疾病预防能力和医疗保障水平显著提升。城乡文化设施进一步完善，文化事业和文化产业加快发展，沧州武术、吴桥杂技被列入国家级非物质文化遗产名录。人口与计划生育工作不断加强，人口自然增长率控制在7.02‰以内。科技创新能力进一步增强，五年累计获得省级以上科技成果39项，建立生产力促进中心18个。加强安全生产和食品药品监管，认真做好社会矛盾纠纷排调工作，有效化解信访隐患，和谐发展的基础进一步巩固。加大审计、监察工作力度，严肃查处了一批违法违纪典型案件。深入开展国防动员和双拥共建活动，军政军民团结不断加强，连续两届被评为全国双拥模范城。民族宗教工作成绩突出，被国务院授予民族团结进步模范集体称号。人防工作扎实推进，连续两届荣获全国人防先进城市。档案、气象、外事侨务、广播电视、妇女儿童等各项事业都取得了新的成绩。

各位代表，五年发展成就的取得，得益于省委、省政府和市委的正确领导，得益于市人大、市政协和社会各界的鼎力支持，得益于全市广大干部群众的共同努力。在此，我代表沧州市人民政府，向辛勤工作在各条战线上的全市各族人民，向给予我们支持与监督的各级人大代表、政协委员和各民主党派、工商联、无党派人士，向大力支持我们工作的离退休老干部、驻沧单位、人民解放军和武警官兵，向所有关心、支持沧州发展的同志们、朋友们，表示崇高的敬意和衷心的感谢！

二、“十二五”时期的指导思想和战略任务

“十二五”时期，是我市强力推进沿海强市建设、打造环渤海地区发展的增长极和隆起带的关键时期。加快沧州经济社会发展，实现富民强市目标，既面临重大机遇，又面临严峻挑战。一是中央经济工作会议指出，我国仍处于加快发展的重要战略机遇期，经济发展长期向好的趋势没有改变，中央将进一步实施积极的财政政策，继续保持宏观经济政策的连续性、稳定性，这为我市发展提供了良好的大环境。二是环渤海地区加速崛起，秦唐沧沿海发展规划即将纳入国家发展战略，沧州的战略地位和竞争优势将进一步提升。三是随着黄骅综合大港的顺利通航，多条高速公路的建成通车，特别是今年京沪高铁的通车运营，沧州将真正进入京津一小时经济圈，后发优势进一步凸显，为我们加快发展增添了新的更大动力。四是一批大项目、好项目先后开工建设或竣工投产，不仅壮大了经济实力、提升了发展层次，而且积蓄了发展后劲，为我们加快发展奠定了坚实基础。五是全市上下浓厚的发展氛围，科学发展、加快发展的良好态势，为沧州加速崛起提供了强大的动力支持。

与此同时，我市发展也面临诸多挑战和压力。从外部环境看，世界经济增长速度减缓，全球需求结构出现明显变化，国际贸易保护主义抬头，人民币升值压力加大，发展的外部环境更加复杂。从国内来看，为应对通胀压力，中央实施稳健的货币政策，贷款投放趋于收紧。同时，国家更加重视资源节约和环境保护，节能减排压力不断加大。从沧州看，经济总量小，结构性矛盾突出，自主创新能力弱，土地、资金、人才、水资源等要素制约较为严重；财政增收难度加大，一般预算收入占全部财政收入比重偏低，收支矛盾突出；一些涉及群众切身利益的问题还没有解决好,改善民生的任务十分繁重。

推进沧州科学发展，不断提高全市人民的生活水平，是我们的第一要务，更是我们义不容辞的责任。必须牢牢把握加快发展、加速转型、率先崛起这个主基调，抢抓机遇，加快沿海强市建设。根据中共沧州市委关于制定“十二五”规划的建议，“十二五”时期我市经济和社会发展的指导思想是：高举中国特色社会主义伟大旗帜，以邓小平理论和“三个代表”重要思想为指导，深入贯彻落实科学发展观，围绕建设沿海强市目标，以加快转变经济发展方式为主线，坚持科学发展不动摇、工业强市不动摇、项目建设不动摇、对外开放不动摇，全力构筑大港口、大产业、大城市三大平台，突出抓好沿海经济龙头带动、城市化建设提质提速、县域经济晋级升位三项重点工作，加快新型工业化、新型城市化和农业现代化进程，努力实现沧州经济社会发展的新跨越。

今后五年，我市经济社会发展的主要目标是：到“十二五”末，实现“三个翻番、四个提高”。“三个翻番”：即经济总量翻一番，地区生产总值突破4000亿元；财政收入翻一番半，达到660亿元；固定资产投资翻一番半，累计完成1.2万亿元。“四个提高”：即新型工业化、新型城市化水平、生态环境质量和人民群众幸福指数明显提高。规模以上工业增加值达到1600亿元；城市化率达到54%；生态体系建设取得明显成效，节能减排完成控制性指标；城市居民人均可支配收入和农民人均纯收入分别达到27000元和9300元，城镇登记失业率控制在4.5%以内，高中阶段教育毛入学率达到85%，城镇职工基本养老保险覆盖人数达到68.4万人，新农合参合率达到95%。

为实现上述战略目标，今后五年将重点推进六大战略任务：

紧紧抓住秦唐沧沿海发展规划即将纳入国家发展战略的机遇，用足、用好相关政策，加快以渤海新区发展为龙头的沿海地区开发开放步伐，早日把沧州打造成为冀中南和环渤海地区新的增长极和隆起带。

加快推进黄骅综合大港建设。坚持政府主导、市场运作、多元开发、加快建设，积极推进20万吨级航道和油品、集装箱、煤炭、矿石等专业码头建设，构筑以综合大港为龙头的集疏运体系，加快综合保税区、出口加工区、保税物流区建设，使黄骅综合大港真正成为环渤海地区具有重要战略地位的现代化综合性大港，充分发挥对全市经济社会发展的拉动作用。

加快推进临港产业聚集。大力发展石油化工、装备制造、电力能源、仓储物流等临港产业，全力打造石油化工基地、装备制造业研发转化基地、电力能源基地和区域性物流中心。加快渤海新区冀中南工业区、沧州临港经济技术开发区、装备制造、冶金和仓储物流等产业园区建设，着力构筑“一区多园”的发展格局。

加快黄骅生态新城建设。按照“先东西、后南北、大聚合”的思路，突出“水为魂、绿为根”的特色，推动主城区、港城区及组团城区协调发展，加快道路交通、商贸服务、休闲娱乐、教育医疗等基础设施和服务设施建设，打造现代化滨海新城。

加快推进港口与内陆腹地互动发展。以基础设施建设为先导，加快完善路网体系，重点打造“沧—衡—邢—邯”、“沧—石—晋—陕”、“ 沧—保—张—蒙”三条发展轴。建立完善港口腹地交流协调机制，推进区域间战略合作，加速形成海陆互动、沿线开发和协调发展的格局。

（二）加快发展方式转变，推进产业结构优化升级。坚持用高新技术改造提升传统产业，加快产业结构优化升级，大力发展战略性新兴产业和高新技术产业，提升我市优势产业的市场竞争力。

做大做强三大支柱产业。石油化工产业：按照“油头化身”的思路，加快推进华北石化1000万吨炼油、中海油中捷石化和沧州炼油厂炼油质量升级和安全环保技术改造等项目，力促“十二五”末达到3000万吨炼油能力。谋划建设100万吨乙烯、100万吨芳烃、100万吨PX、100万吨PVC、30万吨TDI、30万吨MDI、40万吨己内酰胺等项目，构筑以石油化工为主导，现代煤化工、新型盐化工、化工新材料、精细化工“五化并举”的产业集群，全力打造环保、安全、国际化沧州石油化工城。管道装备产业：以重点管道装备企业为依托，以高端、高压、高附加值为方向，上规模、上装备、上水平，到“十二五”末，形成3000万吨加工生产能力，打造国内外知名的管道装备之都。冶金产业：抓住国家和全省钢铁产业向沿海转移的机遇，发挥沿海临港优势，创造条件，吸引更多冶金企业和项目向渤海新区聚集，打造冀中南装备新材料制造基地。在做大做强三大支柱产业的同时，加快机械制造、食品加工、纺织服装等传统产业升级改造步伐。

培育发展战略性新兴产业。坚持重点突破、吸纳承接、融合提升，大力发展电子信息、生物医药、新材料、新能源等战略性新兴产业，形成京津技术成果转化基地和产业对接基地。

大力发展现代服务业。坚持传统服务业与新兴服务业、生产性服务业与生活性服务业并举，加快发展现代物流、金融保险、文化娱乐、休闲旅游、节庆会展、现代商贸、房地产等服务业，提高服务业对经济增长的贡献率。

加快发展现代农业。以优质、高效、外向、生态、安全为目标，以现代科技为支撑，大力发展设施农业，推进畜牧、蔬菜、果品、棉花等特色农产品向规模化、集约化、标准化方向发展。加快农民专业合作组织建设，打造一批产业关联度高、带动能力强的龙头企业，实现农业增效、农民增收。

（三）加快新型城市化步伐，促进城乡一体化发展。以提高城市化水平为主线，以提升城市综合承载能力、引导农村人口合理有序向城镇转移为重点，以建设新城、新区和发展产业聚集区为载体，充分发挥政府规划引导与市场配置资源的双重作用，促进城市化与经济社会发展相适应。

加快建设中心城市。按照沧州市总体规划，谋划实施一批有品位、生财富、惠民生的城建项目，推进城市建设上规模、上层次、上水平。加大开发区、高新区、工业园区建设力度，促进产业聚集，做大做强中心城市经济。加快市区与沧县、青县同城化步伐，进一步扩大主城区规模。完善路网体系，实现中心城区主干道与环城高速对接，所有县通高速公路，促进沧州市区与各县（市、区）的互动、协调发展。

加快发展两个副中心城市。加快任丘市和黄骅新城建设，进一步拉大城市框架，扩大城区规模，提高承载能力，塑造城市特色，打造两个百万人口的区域性中心城市。

加快县城、小城镇和新民居示范村建设。加快泊头、河间两市和盐山、献县等8个县城建设，进一步完善功能，提升品位，扩大规模，增强实力。坚持统一规划、分类指导、示范带动、逐步实施，推进小城镇和农村新民居示范村建设，打造一批产业集聚、人口集中、功能完善、生态文明的特色镇、示范村。

统筹城乡一体化发展。科学编制和完善城乡一体化规划体系，促进城乡各级各类规划的相互衔接和协调，推动城市基础设施、公共服务和现代文明向农村延伸，促进城乡共建、城乡联网、城乡共享。统筹城乡人口流动和劳动就业，逐步建立城乡统一的人力资源市场和户籍管理制度，推动农村人口向城镇有序转移。

（四）加快改革开放，进一步增强发展的动力和活力。深化关键领域和重点环节的改革。深化关键领域和重点环节的改革。行政体制改革：进一步转变政府职能，强化社会管理和公共服务，提高行政效能，为各类企业在我市发展创造优良的环境；加快发展非公有制经济，鼓励大、中、小各类规模的民营企业共同发展，推动民营企业在一、二、三产业中的各个行业竞相发展。财税体制改革：优化财政收入结构，着力增加一般预算收入；优化财政支出结构，加快构建公共财政体系，把新增财力更多用于农业、科技以及文化、教育、卫生、社会保障等公共服务领域。企业改革：加快市属国有、集体企业改革步伐，转换管理体制，优化经营机制，盘活闲置资产，积极推进优势国有企业的公司制改造和企业集团组建步伐。医药卫生体制改革：按照保基本、强基层、建机制的要求，在重点改革任务上取得突破。农村综合配套改革：积极推进农村产权制度改革，深化集体林权制度改革，支持农民专业合作社和农业产业化经营发展。文化体制改革：创新公益性文化事业和经营性文化产业的体制机制，推进经营性文化单位转企改制，整合杂技、武术资源，组建杂技、武术企业集团。

推进全方位、多层次对内对外开放。加强与京津全面对接。积极开展与国家部委、金融机构、科研院所和外国驻华商务机构的对接活动，推进与京津在基础设施、产业发展、高端人才、信息网络、金融服务等方面的交流与合作，承接京津产业转移。积极引进战略投资者。推进与世界500强、国内500强等大企业、大集团的交流合作，在战略合作、战略重组上取得更大突破。搭建对外开放平台。充分发挥工业聚集区、开发区对外开放的窗口作用，完善功能，强化服务，增强对项目、资金等要素资源的吸纳能力。发挥杂技、武术文化资源优势，以杂技节、武术节等节庆活动为平台，积极开展经贸洽谈活动，提高沧州的知名度和对外影响力。

（五）加快保障能力建设，构筑经济发展的战略支撑。进一步完善交通、能源、水利等基础设施，增强科技、人才有效供给能力，为加快发展提供有力支撑。

进一步构筑交通支撑。按照“东出西联、南北贯通、内网加密、外部对接”的原则，加快推进沿海、邯港、石港等高速公路和邯黄铁路建设，完善国省干道和农村公路网。加快推进沧州机场前期谋划，建设冀中南地区重要的支线机场。

进一步构筑能源支撑。加快建设沧东电厂三期、华润热电联产项目，谋划建设核电、海上风电等新能源项目，打造京津冀地区重要的电力能源基地。加强与神华集团以及山西、内蒙古等地区的沟通协作，提高煤炭供应能力。加强与中石油、中海油、中石化等企业的合作，谋划建设商业性原油储备基地，提高油气保障能力。

进一步构筑水利支撑。抓住国家加大对水利设施投入力度的机遇，完善优化水资源战略配置格局，提高水资源调控水平和供水保障能力，促进水利可持续发展。结合引黄济津和南水北调东线工程，谋划实施区域调水工程，加快沧州市区第二水源地、杨埕水库二期等10大引蓄水工程，以及农田水利基础设施和防洪保安全工程建设，形成“东西连通、南北互济、库渠互补”的生态水系水网。

进一步构筑科技、人才智力支撑。围绕全面提高自主创新能力，发挥政策引领作用，构筑以企业为主体、以市场为导向、产学研紧密结合的科技创新体系。推进实施人才引进计划，健全配套政策，完善服务机制，真正做到人才引得进、留得住、用得好。

（六）加快发展民生和社会事业，着力构建和谐沧州。坚持以人为本，加大社会事业投入力度，积极推进公共服务均等化，让全市人民尽享改革发展成果。

进一步完善社会保障体系。完善城镇基本养老、医疗、失业、工伤、生育等保险体系和城乡居民最低生活保障制度，努力构建人员范围广覆盖、保障水平多层次、制度办法可衔接、管理服务现代化的全民社保体系。加大廉租住房和经济适用住房建设力度，着力提高住房保障能力。建立城乡居民收入与经济发展同步增长的机制，不断提高城乡居民收入水平。

努力扩大和促进就业。实施更加积极的就业政策，不断完善就业服务体系，鼓励支持自主创业，多渠道开发就业岗位，提高劳动者就业能力，促进城乡劳动者充分就业。

构建完善的教育体系。坚持把教育放在优先发展的战略地位，全面贯彻落实国家、省《中长期教育改革和发展规划纲要》精神，加大教育改革力度，积极开展各类教育改革试点工作。加快发展学前教育，全力推进义务教育均衡发展，加快普及高中阶段教育，大力发展职业教育，建立完善高等教育学科专业体系和人才培养体系，谋划建设一所综合性大学，为沿海强市建设提供人才支撑和智力支持。

加强医疗卫生和计划生育工作。健全覆盖全市的疾病预防控制、卫生监督和医疗救治网络体系，提高公共卫生服务水平和应对突发公共卫生事件的能力。加强城乡医疗卫生服务体系建设，新增医疗卫生资源重点向农村和城市社区倾斜。巩固和完善新农合制度，积极稳妥地推进公立医院改革，满足城乡群众基本医疗需求。坚持稳定计划生育政策，控制人口数量，提高人口素质。

繁荣发展文化体育事业。加快建设杂技和武术两大场馆、两大学院、两大演出集团，培育两大节庆品牌，打造吴桥杂技大世界和沧州武术城两大基地，推动杂技、武术文化产业上规模、上层次、上水平。加大政府投入，完善公共文化体育设施，繁荣发展文学艺术、新闻出版、广播影视等文化事业，全力办好第十四届省运会，建设文化强市、体育名市。

加强和创新社会管理。积极推进民主法制建设，创新机制、提高能力、完善格局，健全群众权益维护机制，建立调处化解矛盾纠纷平台，加大公共安全投入，完善预防预警和应急处置体系，切实维护社会和谐稳定。

各位代表，“十二五”将是沧州更快更好发展的五年，我们要抢抓机遇，加快发展。我们坚信，在省委、省政府和市委的坚强领导下，全市710万人民团结奋斗、合力攻坚，今后五年的宏伟目标一定会实现、也一定能够实现，一个更具实力、活力、竞争力的新沧州必将崛起于渤海之滨！

三、2011年经济社会发展的重点工作

今年是“十二五”的开局之年，起好步、开好局至关重要。我们必须以科学发展为主题，以转变经济发展方式为主线，以“稳增长、调结构、惠民生、促和谐”为主攻方向，抢抓机遇，强力推进沿海经济龙头带动、城市化建设提质提速、县域经济晋级升位，实现经济社会平稳较快发展，以优异成绩向建党90周年献礼。

今年经济社会发展的主要预期目标是：地区生产总值增长12%以上；固定资产投资增长25%以上；全部财政收入增长15%以上，地方一般预算收入增长15%以上；城镇居民人均可支配收入增长10%，农民人均纯收入增长10%。

重点抓好以下十个方面的工作：

（一）着力抓好渤海新区开发建设。抓住秦唐沧沿海规划即将纳入国家发展战略的机遇，全力推进以渤海新区为龙头的沿海经济发展，为渤海新区地区生产总值在“十二五”实现翻两番的目标奠定坚实基础。一是加快黄骅综合大港二期工程建设。确保20万吨级航道工程和相关专业码头3月份逐步开工，如期建成。二是加快港口配套设施建设。按期建成航运服务中心、海关大厦、检验检疫大厦，完善查验机构办公、生活服务设施；做好综合保税区、仓储物流区前期工作，为实现大通关创造良好的外部条件。三是加快产业聚集。重点推进中捷石化升级改造、北汽40万辆整车、三和重工100万吨大无缝钢管、华润热电2×30万千瓦发电机组等一批产业支撑项目。四是加快黄骅生态新城建设。重点推进企业总部、河北农大渤海校区、北京中医药大学东方学院、国际文化创意广场、博海公园和世博大观园等工程建设。五是加快港区基础设施建设。重点抓好污水处理厂、危险废物处置中心等生产性服务设施建设，积极推进教育、医疗、商业、文化、道路、供水等生活性服务设施建设，保证综合大港运营需要。六是加快港口集疏运体系建设。全力推进邯黄铁路建设，确保沿海高速公路建成通车，滨海公路改扩建工程开工建设。

（二）着力推进重点项目建设。一是抓谋划。重点谋划100万吨乙烯、100万吨PX、30万吨MDI、120万吨醋酸等一批重大产业项目；国华沧东电厂三期、渤海新区热电二期、沧州机场等一批重大基础设施项目。抓住国家和省编制“十二五”规划的机遇，争取更多项目列入省和国家项目“盘子”。二是抓前期。加快推进中捷石化炼油质量升级改造、正元集团醇胺联产、旭阳化工产业园、美国嘉吉粮油储备加工、石钢搬迁等一批重大产业支撑项目，以及邯港、石港高速公路等一批重大基础设施项目前期工作，争取尽快开工建设。三是抓建设。加快推进华北石化1000万吨炼油和沧炼炼油质量升级改造、大化10万吨TDI、金牛化工40万吨PVC、旭阳化工20万吨己内酰胺、宏润重工5万吨压力机组、鑫源泰X100高钢级大口径钢管、华洋660高钢级厚壁直缝焊管等一批重大产业项目建设，确保完成年度建设计划。

（三）着力做大做强工业经济。一是推进开发区、工业聚集区建设。抓住我省推进开发区、工业聚集区、高新区建设的有利契机，加大投入和建设力度，在全市掀起开发区、工业聚集区建设的新高潮。重点编制完善开发区、工业聚集区发展规划，进一步明确产业定位，加快道路、供水、供电、供气、供热等基础设施建设，完善公共服务设施，尽快引进、开工建设一批重点项目，提高产业聚集能力，把开发区、工业聚集区建设成为科学发展示范区、循环经济示范区和工业经济发展的主要载体。二是培育发展企业集团。大力引进战略投资者，通过重组嫁接等形式，做大企业规模，提升企业层次，做强一批优势企业集团。积极开展企业对标行动，推动企业提档升级、提质增效。大力实施品牌战略，年内争创3个中国驰名商标。三是提高企业自主创新能力。重点扶持沧州开发区、高新技术开发区及各县（市、区）生产力促进中心建设，筛选100家创新能力强、成长性好的企业，实施创新型企业培育计划，培育和发展壮大一批科技创新型龙头企业。确保国家级管道元件检测中心建成达标，力争国家级石油化工检测中心获得批准。四是切实抓好生产运营。加强对重点企业、重点项目、重点产品的监控，认真做好市场预测和预警分析，加强煤电油气运等生产要素协调调度，确保企业正常生产经营。

（四）着力推进城镇建设上水平。一是推进中心城区建设上水平。重点抓好十大城建工程：1、沧州新城公建设施建设工程。加快体育场、图书馆、博物馆、规划馆、市民服务中心、京沪高铁沧州站综合楼等重点工程建设。2、企业总部建设工程。加快推进沧州新城管道大厦、移动通讯枢纽楼、东塑大厦等23个企业总部建设。3、城市交通体系建设工程。抓好吉林大道等7条续建道路建设，开工建设沧青快速路，启动京沪铁路维明路立交桥建设，加快公交枢纽站工程建设。4、住房建设工程。抓好廉租房、公租房、经济适用房等保障性住房建设，推进皇家壹里、兰亭苑等6个精品小区建设。5、“三改”（城中村、旧城区、旧小区改造）工程。加快推进大和庄、大季屯、荷花池区域等10个已完成拆迁的城中村和旧城区建设，启动王庄子、十三化建等24个城中村和旧城区拆迁改造。6、“四化”（绿化、美化、亮化、净化）及运河景观改造工程。着力抓好名人植物园、千童公园、滨河公园、沧北景观带建设，做好京沪高铁沿线、环城路沿线、上海路等路段的绿化工作，以及运河景观带建设。推进重要道路、设施、公园、广场及标志性建筑物的亮化、美化，建设一批生活垃圾收集站、便民公厕等环卫设施。7、民生设施建设工程。重点抓好河北工专、一中新校区建设工程，推进十四中、十五中等5所中学和南环小学、实验小学等6所小学迁建和改扩建。抓好公益性综合殡葬服务项目、社区服务中心、妇女儿童活动中心和青少年宫建设项目。加快沧州中西医结合医院病房楼建设。8、商贸物流建设工程。重点抓好泰大国际家居博览中心、颐和新世界、西体育场商业综合体、恒顺世纪中心、荷花池综合购物中心、沧州火车站商业综合体、彩龙国际商贸城、沧州综合物流园区、生产资料物流园、信发商业广场等项目建设。9、集中供热、“两厂”及管网建设工程。启动建设沧州华润热电3号热电联产机组，推进华润运东热电联产项目前期工作，抓好运东污水处理厂升级改造和垃圾发电厂续建工程。铺设和改造供排水、燃气、供热、供电管网172公里。10、防灾减灾工程。重点抓好人防工程、公共消防设施和地震应急避难场所建设。二是推进县城、小城镇和新民居建设上水平。以道路、供水、供热、商贸流通、教育文化等设施建设为重点，加快完善县城功能，提高对要素资源的吸纳能力。发展壮大交河、米各庄等13个重点镇，建设一批与县城相呼应、辐射周边乡村的中心镇、产业镇。进一步抓好新民居示范工程建设，确保一批示范村建成入住。三是推进城市管理上水平。进一步落实“两级政府、三级管理、四级落实”的管理体制，充分发挥两区、街道、社区组织的管理职能。积极创建国家级园林城市、卫生城市、文明城市和环保模范城市。

（五）着力发展现代服务业。一是大力发展物流业。加快渤海新区国际物流园区、沧西物流园区、沧东物流园区以及沧州客运西站物流配送中心等10大物流配送中心建设，构筑融通京津、联通海陆及腹地的现代物流体系。二是大力发展商贸服务业。以建设商业综合体、专业批发市场和引进名品名店为重点，加快京沪高铁沧州站商业广场、颐和广场、一中前街商业广场等大型商业设施建设，搞好沧州火车站站前商业街、小南门商业区等商业设施升级改造，不断发展壮大城市经济。三是大力发展新兴服务业。积极发展文化创意、休闲娱乐、家政服务、医疗健身、电子信息等产业。做大做强沧州银行、沧州建投、渤海投资公司等地方投融资平台，有序发展村镇银行、担保公司和小额贷款公司，积极吸引全国性、区域性银行保险、证券期货、信托担保等各类金融机构和中介机构进驻沧州。四是大力发展旅游业。围绕历史文化、民俗文化、海洋文化和生态休闲四大主题，积极开展与京津等地大型旅游社团的合作，规划、开发、建设精品景区、精品线路，促进我市旅游业快速发展。

（六）着力壮大县域经济和民营经济。一是打造县域经济发展平台。按照确定一个高水平的规划、建设一套较完备的基础设施、培育一批有规模和实力的龙头企业、形成一条较为完整的产业链、组建一个强有力的管理机构“五个一”标准，加快推进工业聚集区和产业园区规范化建设。以县城总体规划和土地利用规划修编调整为契机，依托县城建设工业聚集区、产业园区，重点打造任丘石油化工、盐山孟村管道管件、青县石油装备、泊头数控机床、东光纸箱机械、南皮五金机电、河间电线电缆、肃宁皮草等一批工业聚集区和产业园区，力促每个县（市）都拥有一个省级工业聚集区。二是培育壮大特色主导产业。按照每个县（市）培育壮大1—2个支柱产业的要求，努力做大做强有优势、有潜力的特色支柱产业，重点发展管道装备、数控机床、五金机电、电线电缆、食品加工等特色产业。坚持一手抓增量、一手抓存量，一手抓引进、一手抓改造，打造一批基础条件好、带动能力强的骨干龙头企业，培育一批知名度、美誉度高，拥有自主知识产权的知名品牌。三是大力发展民营经济。认真落实支持鼓励民营经济发展的各项政策措施，按照“非禁即入”的原则，政策上放宽，发展上放手，尤其要着重解决民营企业融资信贷、土地供应、技术人才、管理服务等突出问题。着力发展一批大型企业集团，大力培育和发展中小企业，让民营企业成为吸纳就业、增加税收、活跃经济的生力军。实施中小企业规模倍增、素质提升工程，重点扶持发展有自主专利技术、市场前景好的中小企业，提高经营管理和市场开拓能力。完成市、县人力资源市场建设任务，建立健全政策扶持、创业培训、创业服务三位一体的工作机制，促进全民创业、家庭创业、自主创业，激发群众的创业热情，让更多的劳动者成为创业者。

（七）着力做好“三农”工作。一是调整优化农业产业结构。在保证粮食安全的基础上，加快畜牧水产养殖基地、特色蔬菜基地和优质棉花基地建设，以市场需求为导向，以京津粮油蔬菜市场为重点，大力发展现代设施农业，提高农业生产经营效益。重点建设50个规模化、标准化种植养殖示范基地，100个规模化、标准化养殖场，力争年内全市发展设施蔬菜10万亩以上。二是做大做强农业产业化龙头企业。重点扶持孟村大成、献县沧华园、青县小洋人、肃宁华斯、河间国欣、泊头万雉园、沧县好想你枣业等50家龙头企业，培育10家以上产值超亿元的产业化龙头企业集团。以龙头企业为引领，优化农业结构，促进规模化经营，实现农业增效、农民增收。三是大力发展农民专业合作组织。坚持把发展农民专业合作组织作为新型农业的主体形式，大力发展各种类型的合作社、专业社，不断提高农民组织化程度，年内新增农民专业合作社500家。重点抓好100个农民合作示范社建设，在强化内部管理，扩规扩面的同时，促进合作经济组织提质增效。四是加强农村基础设施建设。抓住国家进一步加大对“三农”投入、加快水利改革发展的机遇，谋划实施一批农村基础设施项目。建立农业、水利投入稳定增长机制，切实抓好农田水利基本建设。加快农村道路、供水、供电、网络通讯等设施建设，积极发展农村沼气，不断改善农村生产生活条件。深入推进“万村千乡”市场工程，进一步健全农村新型流通网络。

（八）着力深化改革，扩大对内对外开放。充分发挥环京津、环渤海和沿海临港优势，大力实施沿海开放战略。一是全力办好“一节一会”。积极筹备第十三届中国吴桥国际杂技艺术节暨第三届中国沧州管道装备展览会，精心组织好主题活动和配套活动，宣传推介沧州，扩大市场营销，引进项目资金。二是推进与京津特别是央企的全面对接。积极开展与北京、天津的对接活动，推进与央企的战略合作，在引进域外资金、技术、项目上实现更大突破。三是加快创新招商方式。坚持“走出去”与“引进来”相结合，瞄准港台、日韩、欧美等国家和地区，积极开展领导招商、产业招商、园区招商和“一对一”小团组招商，增强招商引资的针对性和实效性。充分发挥企业招商、专业招商的主体作用，进一步健全招商引资机制，提高招商引资的成功率。四是积极发展外经外贸。实施多元化国际市场开拓战略，在巩固传统出口市场的同时，大力开拓新兴市场。支持更多企业从事对外贸易和进出口业务，促进特色产品出口，年内争取被批准为国家级精细化工出口基地。积极扩大劳务输出，鼓励有实力的企业参与海外并购，在利用国际资源、开拓海外市场上迈出新步伐。

认真抓好经济社会领域各项改革。财税体制改革，完善财政收入管理体制和转移支付制度，推动城乡和县（市、区）基本公共服务均等化。深化预算编制改革，加强项目评审论证，推进预算绩效管理，扩大政府采购范围及规模。企业改革，积极推进国有和集体工商企业合作重组、破产改制工作，确保进入破产程序的企业完成破产终结。农村综合配套改革，探索农村产权制度改革，推行房屋及土地承包权抵押，完善农村金融服务，探索组建农业担保公司、投资公司和保险公司，破解农村融资担保难题。医药卫生体制改革，全面启动基层医药卫生体制改革，扩大国家基本药物制度实施范围，建立完善的基本药物供应保障体系，保证群众用药安全，降低群众基本医疗费用支出。

（九）着力抓好节能减排工作。一是大力推进节能降耗。严把新上项目能耗关、环评关，严控新上“双高”项目，推动绿色低碳发展。按照国家产业政策要求，坚决淘汰能耗高、污染重、效益差的生产工艺和产品，积极推进重点企业实施技术改造，确保完成省规定的节能目标。二是加强对重点领域的治理。对各县（市、区）、重点企业实施节能减排目标管理。继续深入实施“8755”减排和“十大节能”工程，突出抓好100家年耗能3000吨标准煤以上的重点企业，全面推进工业、建筑、交通和公共机构等重点领域的节能减排工作。深入实施重点流域环境综合整治工程，严厉查处各类破坏生态环境的违法行为。加强基本农田保护，调整建设用地布局，引导工业用地向开发区、工业聚集区集中，不断提高土地利用率。加大污水处理、垃圾处理“两厂”建设力度，强化管理，提高运营能力。三是大力发展循环经济。加强废渣、废水、废气“三废”的综合整治和综合利用，建立废旧物资和再生资源回收利用体系，发展资源回收利用产业。按照“减量化、再利用、资源化”的要求，加快推进沧州临港经济技术开发区等循环经济试点工作，大力推行清洁生产、循环生产模式，建设一批循环经济示范园区，培育一批循环经济示范企业。

（十）着力实施“十大民生工程”。一是社会保障工程。加快社会保障体系建设，提高社会保险统筹层次。年内基本实现失业保险、工伤保险市级统筹，做好破产企业退休人员参保工作，力争年内各类企业退休人员全部纳入医保范围。积极推进新农合工作，进一步扩大新型农村养老保险覆盖面。加快光荣院、敬老院、福利院建设，加强管理，规范运营，提高集中供养水平。鼓励和支持社会力量参与养老服务事业建设。加大住房公积金归集力度，着力解决职工住房问题。二是就业再就业工程。进一步完善零就业家庭、破产企业失业职工、城镇拆迁失业人员等困难群体托底安置机制。大力抓好大中专毕业生就业工作，确保城镇登记失业率控制在4.5%以内。三是住房保障工程。加大保障性住房筹集、建设力度，落实廉租住房补贴，确保低收入家庭住有所居。四是教育提升工程。积极推进主城区教育设施建设和全市中小学校舍安全工程，大力实施基础教育三年行动计划，推动全市基础教育达标升级，创建标准化初中20所、标准化小学100所。着力抓好学前教育示范园建设。整合职教资源，探索职业教育集团化办学模式。完善高等教育体系，提升高等教育水平。五是医疗健康工程。加快基层医疗卫生服务体系建设，确保如期完成建设任务。推动组织好市、县医疗卫生单位定期下乡服务，加快基层医疗卫生机构人才引进和培养，提升农村医疗卫生服务水平。大力开展优生促进工程，积极做好妇幼保健工作，提高出生人口素质。加强重大传染疾病预防控制，健全完善公共卫生体系和突发公共卫生事件应急机制。六是文化体育建设工程。深入实施县乡文化站、图书馆、农村书屋及科普惠民工程，积极推进全民健身活动，丰富群众性文化、体育生活。以杂技节、武术节“两节”为载体，谋划举办层次较高、特色突出、有影响力的文艺演出和体育竞技活动，进一步发展壮大文化、体育产业。七是饮水安全工程。加快推进东光、吴桥、肃宁、任丘等7个县（市）水库建设前期工作，实施沧州东部地区人饮安全工程，谋划建设市区第二水源地，积极做好引水、蓄水、用水文章，着力解决城乡居民饮水安全问题。八是扶贫开发工程。坚持以项目为载体，以产业为支撑，以整合扶贫资金为手段，大力实施产业扶贫龙头带动工程、扶贫培训工程，加大扶贫资金投入，让更多贫困群众早日脱贫致富。九是市场经济秩序整治工程。严格落实食品、产品质量安全责任制，将食品、产品质量安全监管工作纳入各级政府考核内容。加大食品、产品质量安全整顿和药品安全专项整治力度，开展枣制品、熟肉制品、屠宰加工及汽车摩托车配件、建筑材料、小化工等专项整治活动，严厉打击制假售假行为，保障人民群众生命及财产安全。十是平安沧州创建工程。严格落实安全生产责任制，加大对危险化学品、道路交通、易燃易爆和人员密集场所的监管力度，坚决杜绝重特大安全事故发生。深入推进平安沧州建设，加强法制宣传，强化社会治安综合治理，积极排查化解和妥善处理社会矛盾，深化完善城乡一体化治安巡控体系，营造和谐稳定的社会环境。

大力支持工会、共青团、妇联等群众组织开展工作，积极发展妇幼、老龄、残疾人和红十字会等慈善事业。加强国防建设，做好“双拥”和民兵预备役工作。推动档案、人防、民族宗教、外事侨务、气象、地震等各项事业发展。

四、切实加强政府自身建设

完成未来五年战略任务、实现“十二五”精彩开局，需要全市人民团结一心、共同奋斗，更需要政府工作人员恪尽职守，勤奋工作，真正做到为人民服务，对人民负责，让人民满意。

（一）坚持改革创新，推动科学发展、快速发展。随着环渤海地区的加速崛起、秦唐沧沿海发展规划即将纳入国家发展战略、黄骅综合大港建成通航、支撑沧州发展的基础设施进一步完善和交通条件的进一步便利，为沧州加快发展带来了十分难得的机遇。各级政府和政府各部门要进一步增强发展的使命感、紧迫感，勇于自我加压，敢于自我超越，不断创新发展理念、创新发展模式、创新工作方法，千方百计地多上快上项目，千方百计地引进战略投资者，千方百计地发展民营经济，千方百计地推进科学发展、快速发展，以勤奋努力的工作和发展的实绩，提升沧州的竞争实力和战略地位，提高全市人民的生活水平。

（二）坚持一心为民，全心全意为广大人民群众服务。各级政府工作人员要牢记全心全意为人民服务的宗旨，切实做到权为民所用，加快政府职能转变，完善公共服务体系，将公共资源更多地向民生领域倾斜；做到情为民所系，倾听群众呼声，顺应群众意愿，关心群众疾苦；做到利为民所谋，紧紧围绕人民群众的所思、所需、所盼，满腔热情、尽心竭力地为人民群众办实事、干好事、解难事，努力提高人民群众的幸福感、自豪感。

（三）坚持务实勤政，大力营造真抓实干的浓厚氛围。始终保持奋发有为、只争朝夕的精神状态，大力倡导脚踏实地、雷厉风行、誓争一流的工作作风，不断增强干事创业的主动性、创造性，真正把心思用在谋发展、抓发展上，把精力用到干实事、求实效上。不搞形式主义、不做表面文章，殚精竭虑，奋力开拓，努力创造经得起实践、历史和人民检验的工作业绩。

（四）坚持依法行政，不断推进决策民主化和科学化。强化法制观念，规范行政行为，健全行政权力监督机制，推进行政权力公开透明运行。依法实施行政问责，坚决杜绝行政不作为、乱作为。进一步健全决策咨询制度，努力做到依法决策、科学决策、民主决策。自觉接受市人大及其常委会的法律监督，接受市政协的民主监督，主动听取各民主党派、工商联、无党派人士及社会各界的意见和建议，不断改进政府工作，让政府工作更加科学，更加民主，更加符合人民群众的意愿。

（五）坚持廉洁从政，树立清正廉洁的政府形象。认真落实党风廉政建设责任制，积极推进预防和惩治腐败体系建设，严格遵守法律法规和廉洁从政的各项规定，堂堂正正做人，干干净净做事。加强廉政文化建设和廉政制度建设，建立健全拒腐防变的长效机制，着力从源头上、制度上防治腐败。深入推进政务公开，让权力在阳光下运行，广泛接受社会公众和新闻舆论监督，努力建设团结务实、廉洁高效的人民政府。

各位代表，新的任务光荣艰巨，新的目标催人奋进。让我们紧密团结在以胡锦涛同志为总书记的党中央周围，在省委、省政府和市委的坚强领导下，凝聚全市人民的智慧和力量，抢抓机遇，奋力拼搏，为全面完成“十二五”规划确定的各项任务目标，为早日把沧州建设成为宜居、宜业的现代化沿海强市而努力奋斗！
